# Supplementary material for: Transcriptome variations in hybrids of wild emmer wheat (Triticum turgidum ssp. dicoccoides)
Source: BMC Plant Biol. 2024 Jun 18;24:571. doi: 10.1186/s12870-024-05258-3 (PMC11184805; doi:10.1186/s12870-024-05258-3)
Supplement: Supplementary file 2 — Supplementary Material 2 [file 12870_2024_5258_MOESM2_ESM.docx]

Supplementary Table S1. Adaptors and primers used in MSAP reaction

| Eco-adapter1 | 5' CTC GTA GAC TGC GTA CC 3’ |
| --- | --- |
| Eco-adapter2 | 5’ AAT TGG TAC GCA GTC TAC 3’ |
| Eco-pre | 5' GAC TGC GTA CCA ATT CA 3’ |
| E-ACT | 5' GAC TGC GTA CCA ATT CAC T 3' |
| E-ACA | 5' GAC TGC GTA CCA ATT CAC A 3' |
| E-ACC | 5' GAC TGC GTA CCA ATT CAC C 3' |
| H/M- adapter 1 | 5' GAT CAT GAG TCC TGC T 3' |
| H/M- adapter 2 | 5' CGA GCA GGA CTC ATG A 3' |
| H/M-pre | 5' ATC ATG AGT CCT GCT CGG 3' |
| H/M-TCAA | 5' CAT GAG TCC TGC TCG GTC AA 3' |

Supplementary Table S2**.** Cytosine methylation levels in parental lineages and hybrids

| **Primer**  **combination** | **Parental/hybrid group** | **MH** | **MA** | **F4** |
| --- | --- | --- | --- | --- |
| E-ACC | Average No. of Bands | 118.2 | 156.3 | 161.3 |
|  | Average polymorphic bands | 102.2 | 132.3 | 130.8 |
|  | Average Polymorphism (%) | 86.3 | 84.6 | 81.2 |
|  | standard error | 1.7 | 1.0 | 3.0 |
| E-ACA | Average No. of Bands | 100.8 | 131.7 | 133.7 |
|  | Average polymorphic bands | 80.8 | 98.0 | 99.3 |
|  | Average Polymorphism (%) | 80.3 | 74.4 | 71.0 |
|  | standard error | 1.7 | 2.4 | 0.3 |
| E-ACT | Average No. of Bands | 79.0 | 81.8 | 87.3 |
|  | Average polymorphic bands | 71.4 | 71.8 | 62.0 |
|  | Average Polymorphism (%) | 90.3 | 88.1 | 71.2 |
|  | standard error | 2.1 | 3.3 | 2.2 |
| Average | **Average No. of Bands** | **99.3** | **123.2** | **127.4** |
|  | **Average polymorphic bands** | **84.8** | **100.7** | **97.4** |
|  | **Average Polymorphism**  **(%)** | **85.6** ^α^ | **82.4**  ^β^ | **74.5** ^α β^ |
|  | **standard error** | **1.8** | **2.2** | **1,8** |

α and β note statistical significance against F4. The average polymorphism of all three primers combinations was compared using a simple unpaired T-test for each parental group vs. F4 hybrids. In both cases, the differences between hybrids and parents were statistically significant (p-value = 0.01 between MH parental group and F4 and p-value = 005 between MA parental group and F4)
